# Supplementary figures and images for: Revealing potential interfering genes between abdominal aortic aneurysm and periodontitis through machine learning and bioinformatics analysis
Source: PLoS One. 2025 Aug 26;20(8):e0329592. doi: 10.1371/journal.pone.0329592 (PMC12380325; doi:10.1371/journal.pone.0329592)

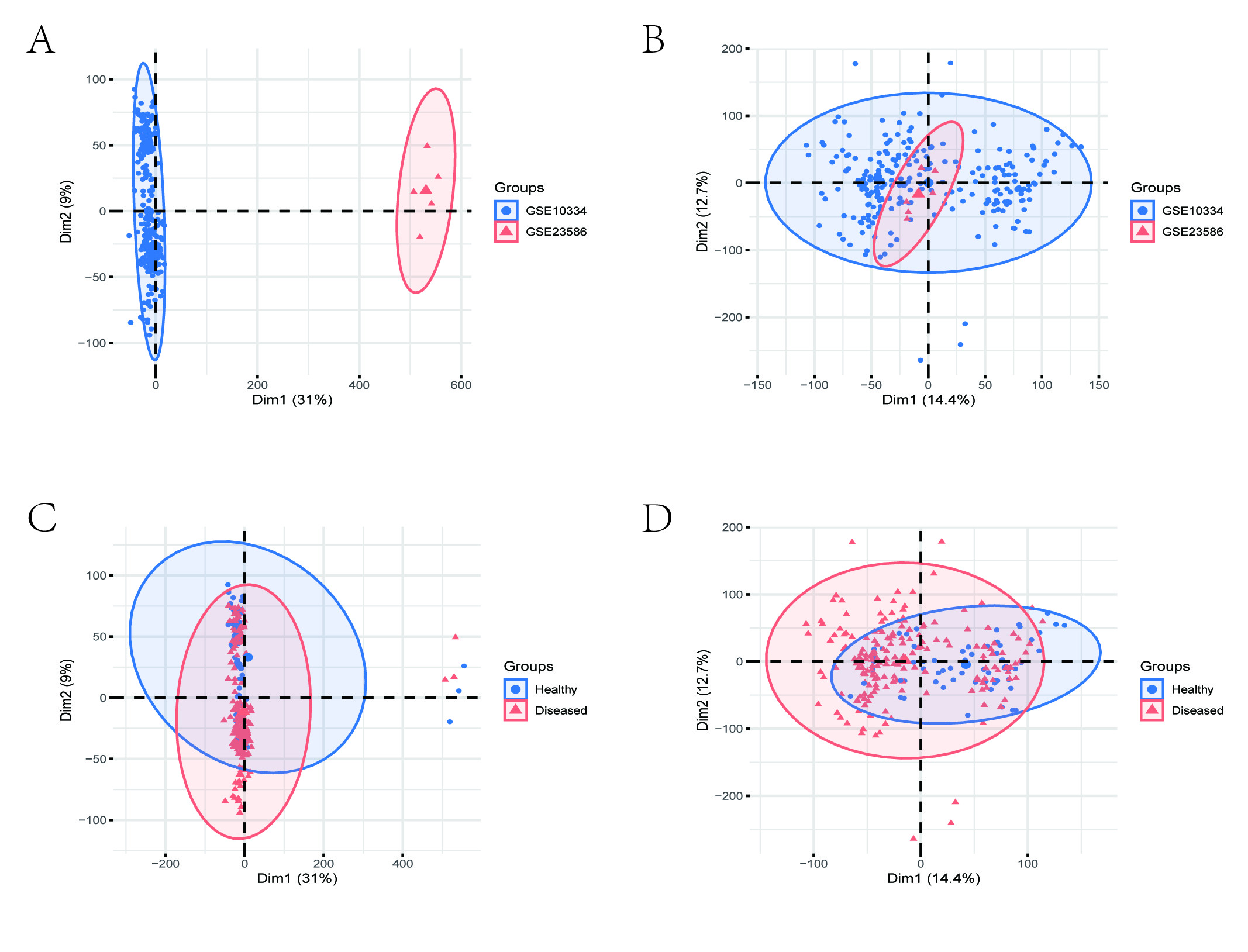

Supplement: S1 Fig — (TIF) [file pone.0329592.s001.tif]

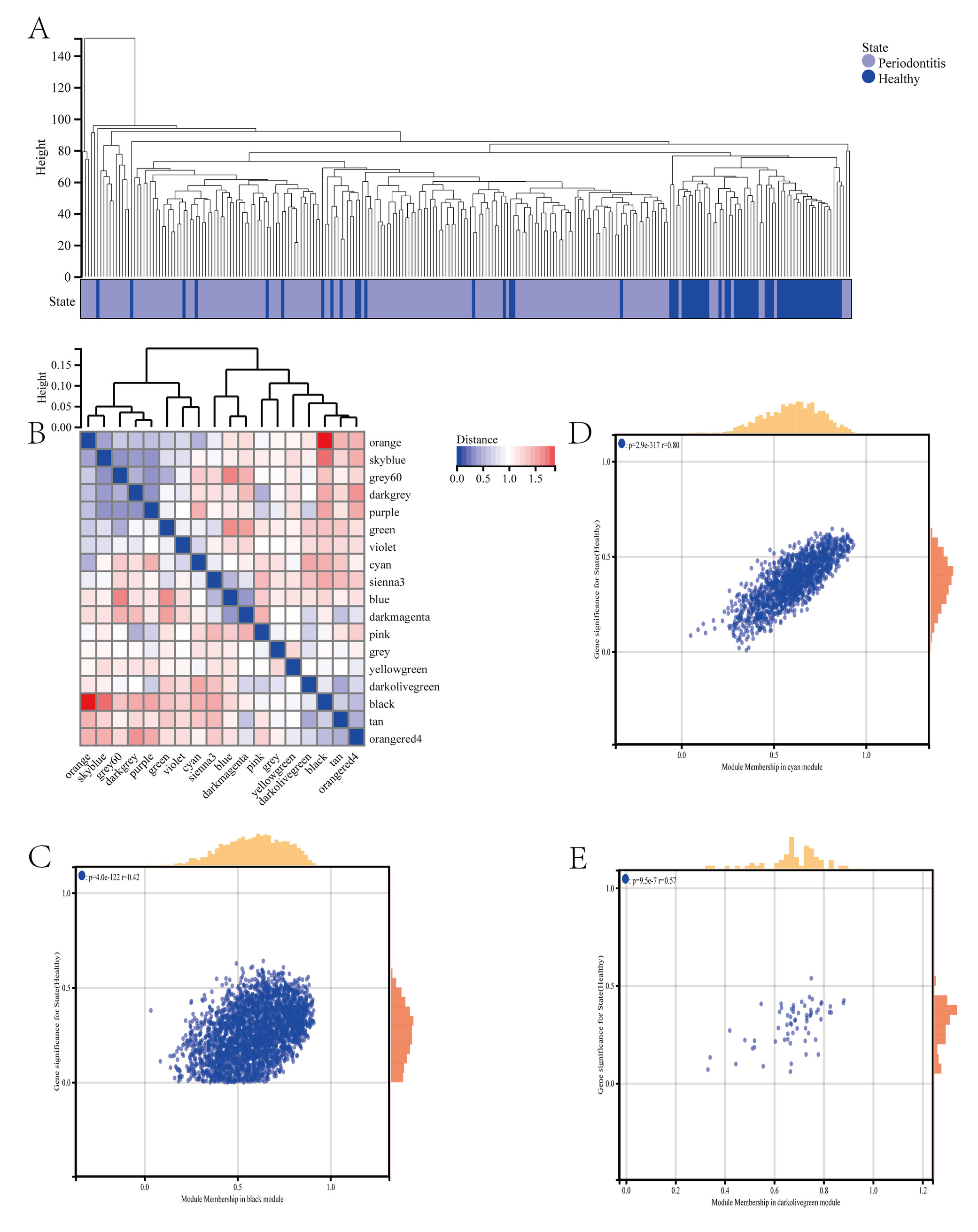

Supplement: S2 Fig — (TIF) [file pone.0329592.s002.tif]

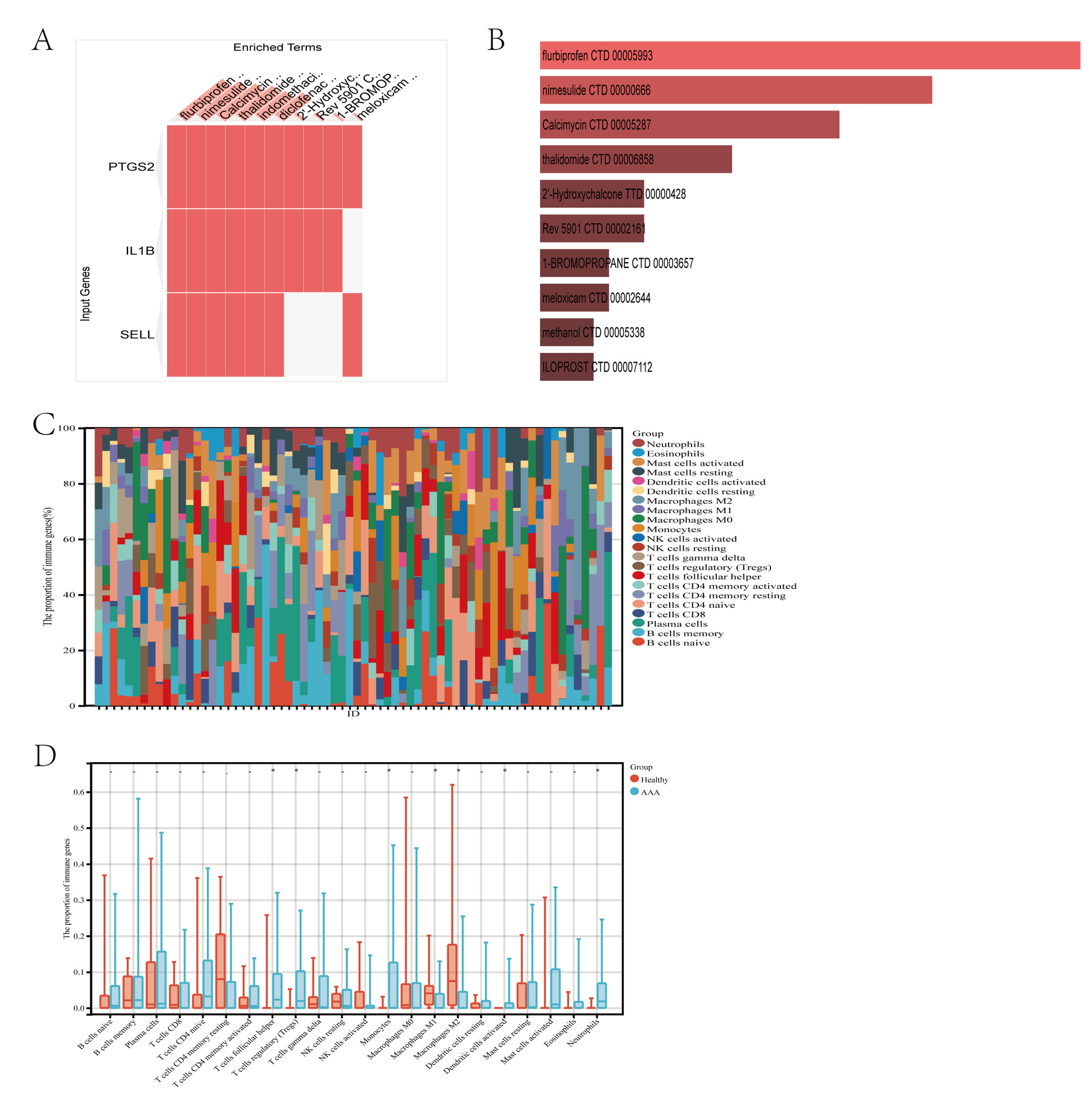

Supplement: S3 Fig — (TIF) [file pone.0329592.s003.tif]
