# Supplementary material for: Revealing potential interfering genes between abdominal aortic aneurysm and periodontitis through machine learning and bioinformatics analysis
Source: PLoS One. 2025 Aug 26;20(8):e0329592. doi: 10.1371/journal.pone.0329592 (PMC12380325; doi:10.1371/journal.pone.0329592)
Supplement: S1 Appendix — All resources are publicly available, with version numbers and access details specified to ensure reproducibility. (DOCX) [file pone.0329592.s004.docx]

**Appendix A: R Packages Used**

Limma (v 3.6.9)

WGCNA (v 1.72-5)

clusterProfiler (v 3.14.3)

org.Hs.eg.db (v 3.1.0)

clusterProfiler (v 3.14.3)

glmnet (v 4.1-8)

caret (v 6.0-94)

randomForest (v 4.7-1.1)

rms (v 6.8-0)

ggplot2 (v3.5.0)

ggvenn (v 0.1.10)

**Appendix B: Software and Websites**

GEO database (https://www.ncbi.nlm.nih.gov/GEO)

R software (version 4.3.3; <https://cran.r-project.org)>

KEGG rest API (https://www.kegg.jp/kegg/rest/keggapi.html)

STRING online database (https://cn.string-db.org; version 12.0)

Enrichr database (https://maayanlab.cloud/Enrichr/)

The code for this study is available on GitHub at https://github.com/yijiayi123123/AAA--P
