# Supplementary material for: Revealing potential interfering genes between abdominal aortic aneurysm and periodontitis through machine learning and bioinformatics analysis
Source: PLoS One. 2025 Aug 26;20(8):e0329592. doi: 10.1371/journal.pone.0329592 (PMC12380325; doi:10.1371/journal.pone.0329592)
Supplement: Attachment — Submitted filename: Rebuttal letter.docx [file pone.0329592.s007.docx]

Dear Editor and Reviewers,

Thank you for your time and thoughtful comments on our manuscript titled “Revealing potential interfering genes between abdominal aortic aneurysm and periodontitis through machine learning and bioinformatics analysis.” We appreciate your constructive feedback, which has greatly improved the quality of the paper. Below, we provide a point-by-point response to each comment. All changes have been incorporated into the revised manuscript for your reference.

**Reviewer 1**

**Response：**Thank you for the valuable suggestions provided by the reviewers. In response to their constructive feedback, I have made several revisions to improve the clarity and comprehensiveness of the manuscript. Specifically, I have moved the detailed descriptions of the software package and tools to the appendix to ensure a more streamlined presentation of the main content. Additionally, I have included comprehensive summary tables in Section 3, which should facilitate easier access for readers to key data and results.

Furthermore, I have removed redundant discussions of the results within the main body of the manuscript. In the discussion section, I have placed greater emphasis on addressing the study's limitations, offering a more thorough examination of aspects such as the necessity for experimental validation, as well as outlining potential avenues for future research. These adjustments aim to provide a more balanced perspective on the findings and their broader implications.

In the conclusion section, I have expanded the content to include a more detailed reflection on the key takeaways from the study, ensuring a more complete synthesis of the findings and their significance.

With regard to the specific request for the AUC and grouping in Figure 7, I have already provided a detailed breakdown of the groupings within the manuscript for clarity and ease of reference. Additionally, as requested, I have made the code used in this study publicly available on GitHub. The repository is now accessible, allowing readers to review and utilize the code as needed for their own purposes.

These revisions aim to enhance the overall quality and transparency of the research, and I hope that the changes address the concerns raised during the review process.

**Reviewer 2**

1. What are the staging and grading of the periodontal cases included in the study? Were there more severe cases among unstable AAA? Any correlation between the severity of periodontitis with AAA diameter and volume?

**Response：**In this study, we did not perform detailed staging or grading of the periodontal cases; they were classified into the periodontal disease group and the healthy control group. Regarding unstable AAA, we did not investigate whether there are more severe cases, and further analysis would be required in this regard. As for the relationship between the severity of periodontal disease and the diameter or volume of AAA, while we did not provide direct correlation analysis in this study, this is a direction worth further investigation, and we plan to explore these potential associations in future research.
2. It is well understood that periodontitis is a site-specific disease. Can the authors provide more periodontal parameters such as probing depth (>6mm) of the cases?

**Response：**In this study, we did not record detailed periodontal parameters, such as probing depth (>6mm), although specific data can be obtained from the GEO database. This represents a limitation of our study. Future research will consider incorporating these more detailed periodontal health parameters to provide a more comprehensive assessment of the severity of periodontitis and analyze its relationship with AAA.

3. Besides of reducing inflammation, what is the clinical implication of this study?

**Response：**We appreciate the reviewer’s question regarding the clinical significance. In addition to exploring the genetic interactions between periodontitis and abdominal aortic aneurysms (AAA), we believe the clinical significance of this study lies in offering a new perspective on the potential systemic effects between periodontal disease and AAA. Periodontitis is recognized as a source of systemic inflammation, and recent research suggests that oral health may influence the onset and progression of other systemic diseases, especially arterial conditions. Although our study did not perform detailed staging or analysis of individual cases, preliminary results suggest that periodontitis may be associated with the formation and progression of AAA, particularly at the genetic level. Therefore, beyond reducing local inflammation, effective management of periodontitis might have a potential clinical impact on the prevention and treatment of AAA. Our research provides a theoretical foundation for future clinical studies and interventions, especially in AAA patients, where there may be a need for greater emphasis on oral health and the management of periodontitis.

We believe that these revisions have addressed all the points raised by the reviewers. Once again, thank you for your valuable comments, and we hope that the revised manuscript meets your expectations. We look forward to your feedback.

Yours sincerely ,

 Liming Tang

 E - mail : tanglm@usx.edu.cn
