# Supplementary material for: Revealing potential interfering genes between abdominal aortic aneurysm and periodontitis through machine learning and bioinformatics analysis
Source: PLoS One. 2025 Aug 26;20(8):e0329592. doi: 10.1371/journal.pone.0329592 (PMC12380325; doi:10.1371/journal.pone.0329592)
Supplement: Attachment — Submitted filename: PONE-D-24-45155_comments.docx [file pone.0329592.s006.docx]

This explores the molecular connections between abdominal aortic aneurysm (AAA) and periodontitis using an integrated bioinformatics and machine learning approach. The authors identify three key overlapping genes (IL1B, PTGS2, SELL) and demonstrate their association with immune infiltration and inflammation, suggesting a potential comorbidity mechanism. The work is timely, well-structured, and makes good use of publicly available transcriptomic data. However, the manuscript requires significant revisions to improve clarity, rigor, and interpretative depth before it can be considered for publication.

1. While the concept of gene overlap in AAA and periodontitis is novel, the mechanistic insight into how these genes contribute to shared pathology is somewhat speculative. Strengthen the discussion by proposing specific mechanisms or citing functional studies related to these genes in both diseases.
2. The immune infiltration results are intriguing but need more context. For example, a significant negative correlation with Tregs is mentioned, but the functional consequence is not explored. Expand on how Treg and neutrophil dysregulation may drive comorbid disease processes, using relevant literature.
3. Integrating transcriptional regulatory network analysis or causal inference methods (e.g., upstream regulator analysis) could clarify whether IL1B is a driver rather than a passenger of inflammation.
4. The KEGG and GSEA results are informative but underdeveloped in the discussion. Include a schematic or proposed model illustrating how these pathways converge via the three key genes to promote comorbidity.
5. Drug prediction via Enrichr is promising but remains descriptive. Discuss the therapeutic relevance of the top-predicted drugs (e.g., flurbiprofen, meloxicam) in the context of AAA or periodontal disease. Is any preclinical or clinical evidence available?
6. Ensure all figures are appropriately referenced and legible. (e.g. Fig. 8C-E)
7. The manuscript would benefit from professional language editing to improve clarity and readability. There are frequent issues with awkward phrasing and redundancy.
