# Supplementary material for: Revealing potential interfering genes between abdominal aortic aneurysm and periodontitis through machine learning and bioinformatics analysis
Source: PLoS One. 2025 Aug 26;20(8):e0329592. doi: 10.1371/journal.pone.0329592 (PMC12380325; doi:10.1371/journal.pone.0329592)
Supplement: Attachment — Submitted filename: Response to Reviewers.docx [file pone.0329592.s008.docx]

Manuscript ID: PONE-D-24-45155R1

Title: Revealing potential interfering genes between abdominal aortic aneurysm and periodontitis through machine learning and bioinformatics analysis

Dear Academic Editor and Reviewers,

We sincerely appreciate your insightful feedback, which has significantly strengthened our manuscript. We have implemented all suggested revisions and provide a point-by-point response below. All revisions are documented in the tracked-changes manuscript, and data/code remain accessible on GitHub (https://github.com/yijiayi123123/AAA--P).

Response to Academic Editor

Language Editing:

We have engaged a professional scientific editing service to refine grammar, syntax, and clarity throughout the manuscript (e.g., Introduction: Lines 35–42; Discussion: Lines 215–230).

Figure Resolution Clarification:

We wish to clarify that all figures were originally created in vector format (300 dpi) and meet PLOS ONE’s standards. No regeneration was necessary.

Enhanced Biological Insights:

We expanded mechanistic discussions per Reviewer 3’s suggestions (detailed below).

Response to Reviewer 1

Parameter Reporting:

We added explicit details to Methods 2.6:

LASSO: 10-fold CV (5 repeats), λ via min BIC (Lines 110–115).

SVM-RFE: Feature elimination to 9 genes, C=1 (Lines 116–118).

WGCNA: deepSplit=2, merge threshold 0.25 (Lines 90–95).

Figure 7 Group Labeling:

We respectfully clarify that existing annotations (Table 2 + figure legends) already distinguish test/validation cohorts and disease groups. To avoid redundancy, we have not modified the figure but enhanced the legend (Page 23).

Response to Reviewer 3

Mechanistic Insights:

We deepened discussions with experimental evidence:

IL1B: Drives VSMC apoptosis (AAA, Ref 52) and osteoclast activation (periodontitis, Ref 51; Discussion Lines 238–245).

PTGS2: Regulated by miR-15b-5p/ACSS2 in AAA (Ref 56); mediates bone resorption via PGE2 (Ref 58, 60).

SELL: Promotes neutrophil adhesion (Ref 63) and correlates with periodontitis severity (Ref 65).

Immune Context:

Added functional interpretations:

Treg suppression deficit → Uncontrolled macrophages (AAA) and osteoclasts (periodontitis) (Lines 255–260).

Neutrophil NETosis drives matrix degradation in both diseases (Lines 265–270; Refs 78–81).

Drug Relevance:

Discussed flurbiprofen’s topical benefits (periodontitis, Ref 60) vs. systemic risks (AAA, Ref 82), proposing canakinumab for comorbidities.

General Revisions

Added Table 3 (gene-drug interactions) to main text (Page 27).

Included 12 new references (Refs 51, 52, 56, 58, 60, 63, 65, 78–83).

We thank the reviewers for their rigorous engagement and hope these revisions satisfy all concerns.

Sincerely,

Liming Tang, Ph.D.

Corresponding Author: tanglm@usx.edu.cn
